# Genetic Variants in MARCO Are Associated with the Susceptibility to Pulmonary Tuberculosis in Chinese Han Population

**DOI:** 10.1371/journal.pone.0024069

**Published:** 2011-08-23

**Authors:** Mai-Juan Ma, Hai-Bing Wang, Hao Li, Jun-Hai Yang, Yan Yan, Lan-Pin Xie, Ying-Cheng Qi, Jun-Lian Li, Mei-Juan Chen, Wei Liu, Wu-Chun Cao

**Affiliations:** 1 State Key Laboratory of Pathogen and Biosecurity, Beijing Institute of Microbiology and Epidemiology, Beijing, People's Republic of China; 2 Department of Tuberculosis, Shijiazhuang Fifth Hospital, Hebei, People's Republic of China; 3 Physical Examination Department, Beijing Electronic Hospital, Beijing, People's Republic of China; 4 Department of Tuberculosis, Hebei Chest Hospital, Hebei, People's Republic of China; 5 Xinjiang Uygur Autonomous Region Chest Hospital, Urumqi, People's Republic of China; Hopital Raymond Poincare - Universite Versailles St. Quentin, France

## Abstract

**Background:**

Susceptibility to tuberculosis is not only determined by *Mycobacterium tuberculosis* infection, but also by the genetic component of the host. Macrophage receptor with a collagenous structure (MARCO) is essential components required for toll like receptor-signaling in macrophage response to *Mycobacterium tuberculosis*, which may contribute to tuberculosis risk.

**Principal Findings:**

To specifically investigated whether single nucleotide polymorphisms (SNPs) in MARCO gene are associated with pulmonary tuberculosis in Chinese Han population. By selecting tagging SNPs in MARCO gene, 17 tag SNPs were identified and genotyped in 923 pulmonary tuberculosis patients and 1033 healthy control subjects using a hospital based case-control association study. Single-point and haplotype analysis revealed an association in intron and exon region of MARCO gene. One SNP (rs17009726) was associated with susceptibility to pulmonary tuberculosis, where the carriers of the G allele had a 1.65 fold (95% CI = 1.32–2.05, *p*_corrected_ = 9.27E–5) increased risk of pulmonary tuberculosis. Haplotype analysis revealed that haplotype GC containing G allele of 17009726 and haplotype TGCC (rs17795618T/A, rs1371562G/T, rs6761637T/C, rs2011839C/T) were also associated with susceptibility to pulmonary tuberculosis (*p*_corrected_ = 0.0001 and 0.029, respectively).

**Conclusions:**

Our study suggested that genetic variants in MARCO gene were associated with pulmonary tuberculosis susceptibility in Chinese Han population, and the findings emphasize the importance of MARCO mediated immune responses in the pathogenesis of tuberculosis.

## Introduction

Tuberculosis (TB) is caused by infection with *Mycobacterium tuberculosis* (*M.tb*) and poses substantial morbidity and mortality worldwide. It is however known that TB susceptibility is not only determined by the infection status and environment factors but also by the host genetic components, as proved by epidemiological [1], [2], [3], twin [4] and adopt studies [5]. Polymorphisms in the HLA haplotype and in the genes for the vitamin D3 receptor (VDR), solute carrier family 11 member 1 (SCL11A1), interferon gamma (IFN-γ) promoter, mannose binding lectin (MBL), nitric oxide synthase 2 (NOS2A), and some toll like receptor (TLR) genes have all been associated with increased susceptibility to *M.tb*, although individually, the attributable risk of each polymorphism is modest [6]. Therefore, the genetic influence is likely to be polygenic in nature. Many of the identified susceptibility alleles act at the level of the macrophage. *Mycobacteria* are facultative intracellular bacteria that infect and survive in host macrophages, the receptors expressed on the macrophage is initially responsible for detection and recognition of bacilli. Then the activation of receptors causes an immediate signaling transduction resulting in the induction of pro-inflamation cytokines, which are important for resistant or susceptibility to *M.tb* infection.

Scavenger receptor (SR) macrophage receptor with a collagenous structure (MARCO) is a class of phagocytic receptors that has been implicated in host defense against bacterial pathogens [7]. Ito *et.al* found that the MARCO expression was transiently up-regulated on macrophages in response to BCG infection and expressed on macrophages within, and adjacent to, BCG-containing granulomas [8]. MARCO -expressing macrophages in the splenic marginal zone appear to phagocytose more BCG than neighboring macrophages that do not express MARCO [8]. The following study suggested that the MARCO was expressed constitutively on subsets of macrophages and was up-regulated in response to TLR agonists and whole bacteria [9] but not by pro-inflammatory cytokines [10]. In vivo study, MARCO expression increased on macrophages in response to infection or inflammatory conditions [10], [11] and this occurs on macrophages that are directly responsive to the stimuli as well as those distal to the initial infectious stimuli [7], [12], [13]. Consequently, the increased expression of MARCO may alter the function of MARCO-expressing macrophages by increasing bacterial binding and phagocytic capacity and by altering cytokine production [14]. The evidence from the recent study indicated that the MARCO mediated recognition and presentation of trehalose 6, 6′-dimycolate (TDM, also known as cord factor) and the macrophages expressed MARCO receptor secrete pro-inflammatory cytokines in response to TDM. Macrophages from MARCO defective mice also produce significantly lower levels of pro-inflammatory cytokines than wildtype macrophages in response to infection with virulent *M.tb* and identify MARCO might be the additional co-receptors required for TLR-signaling in macrophage response to *M.tb* and TDM [15]. All of these studies so far provided strong evidence that MARCO might also act as an important mediator in the host immunity against *M.tb*.

Given the strong functional role of MARCO in host immunity against *M.tb* infection, the genetic component for MARCO might be conferred resistance or susceptibility to pulmonary TB (pTB). In the present study, we investigated MARCO polymorphisms for association with pTB susceptibility.

## Results

### Demographic characteristics

All of the subjects were of Chinese Han descent population. The baseline characteristics of the study population, including 923 pTB patients and 1033 NHS, are as shown in table 1.

**Table 1:** The demographic characteristics of study population.

Characteristics	Patients	Controls	*p*-value
**Sex**			0.700
Female	314(34.0)	360(34.8)	
Male	609(66.0)	673(65.2)	
**Age yrs**	36.4(16.7)	37.6(13.5)	0.090
**Smoking**			0.075
Smoking	200(21.7)	189(18.3)	
Nonsmoking	681(73.8)	781(75.6)	
Ever smoking	42(4.5)	63(6.1)	
BCG vaccination			0.078*
Yes	185(20.0)	248(24.0)	
No	669(72.4)	738(68.5)	
Uncertain	69(7.6)	77(7.5)	
**Tuberculin skin test**			
Positive	498(54.0)	ND	
Strong positive	332(36.0)	ND	
Negative	44(4.7)	ND	
ND	49(5.3)	ND	
**Clinical phenotype**			
Pulmonary tuberculosis	923		
culture positive	389 (42.1)	ND	
Smear positive	294 (31.9)	ND	
Culture/smear positive	240 (26.0)	ND	

Data are presented as n (%) or Mean (SD), unless otherwise stated. ND: Tuberculin skin test was not determined.

*p value was calculated by BCG vaccination status (yes and no) between two groups.

### Analysis of MARCO SNPs in Chinese Han population

A power of detection>90% was achieved for both multiplicative and additive models, assuming an approximative TB prevalence of 0.007 in China, a frequency of 0.1 for high risk alleles and a genotype relative risk of 1.4 (a = 0.05) with our sample size (case–control ratio = 0.894). Fourteen of the 17 SNPs genotyped were in Hardy-Weinberg equilibrium and three SNPs (rs6751745, rs4491733 and rs12987402) showed deviation from HWE (*p* <0.05, data not shown). The remaining 14 SNPs were further estimated for their association with pTB using Chi-square test. Single-marker analysis indicated that the minor G allele of rs17009726 was over-represented in case subjects than in controls, after the Bonferroni correction for multiple testing of 14 SNPs (*p*_corrected_ = 4.93E–5, table 2), thus showing significant association with pTB. Binary logistic regression analysis was used to identify any possible effect of MARCO genotype on pTB, with sex, age, smoking and BCG vaccination status as covariates. Moreover, the AG genotype was significantly associated with an increased risk of pTB by multivariate analysis. This increased risk was present by both additive and dominant model (OR = 1.61, 95%CI = 1.28–2.01, *p*_corrected_ = 4.96E-4 and OR = 1.65, 95%CI = 1.32–2.05, *p*_corrected_ = 9.27E-5; respectively, table 3).

**Table 2:** Associations between *MARCO* gene allele frequencies and tuberculosis in Chinese Han population.

rs#	Alleles	MA	Case	Control	*p*-value	*p*_corrected_-value
**rs17009242**	G/A	A	0.153	0.151	0.842	
**rs1318645**	C/G	G	0.340	0.359	0.213	
**rs2077344**	C/T	T	0.350	0.367	0.286	
**rs6752783**	T/G	G	0.203	0.210	0.593	
**rs7559955**	C/T	T	0.214	0.224	0.467	
**rs17009726**	A/G	G	0.139	0.092	3.52E-6	**4.93E-5**
**rs2278588**	C/T	T	0.247	0.258	0.451	
**rs17795618**	T/A	A	0.199	0.221	0.086	
**rs1371562**	G/T	T	0.115	0.131	0.134	
**rs6761637**	T/C	C	0.163	0.133	0.0083	0.116
**rs2011839**	C/T	T	0.034	0.033	0.907	
**rs3731611**	G/T	T	0.045	0.041	0.556	
**rs4848530**	C/T	T	0.247	0.264	0.181	
**rs11685286**	T/C	C	0.146	0.137	0.459	
**rs6751745**	C/T	T	0.279	0.251	ND	
**rs4491733**	A/G	G	0.468	0.471	ND	
**rs12987402**	T/C	C	0.404	0.414	ND	

Data are presented as minor allele frequency, unless otherwise stated. MA: minor allele; HW: Hardy-Weinberg equilibrium; OR: odds ratio, CI: confidence interval; ND: p value was not determined due to the probability for deviation from Hardy-Weinberg equilibrium in controls and cases.

**Table 3:** Multivariate logistic regression analysis of rs17009726A/G genotype in pulmonary tuberculosis cases and controls.

Model	Genotype	Case	Control	OR (95% CI)	*p*_adjusted_-value	*p*_corrected_-value
Cod	AA	0.743	0.827	1.00		
	AG	0.235	0.163	1.61(1.28–2.01)	**3.54E-5**	**4.96E-4**
	GG	0.022	0.011	2.26(1.08–4.76)	**0.031**	0.434
Dom	AA	0.743	0.827	1.00		
	AG-GG	0.257	0.173	1.65(1.32–2.05)	**6.62E-6**	**9.27E-5**
Rec	AA-AG	0.978	0.989	1.00		
	GG	0.022	0.011	2.06(0.98–4.32)	0.051	0.714
Add				1.57 (1.29–1.92)	7.86E-6	**1.10 E-4**

Data are presented as frequency of genotype, unless otherwise stated; Cod: codominant model; Dom: dominant model; Rec: recessive model; Add: additive model; OR: odds ratio; CI: confidence interval.

**Figure 1:**
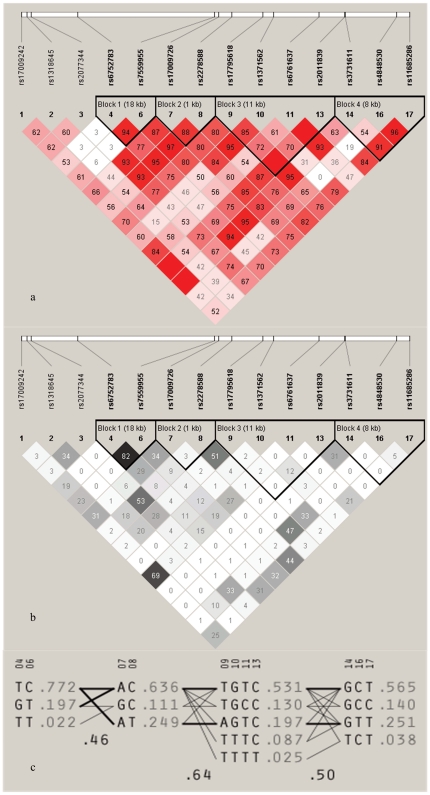
Linkage disequilibrium (LD) map of SNPs. (a) LD map based on D-prime (D’); (b) LD map based on R-squared (R^2^); (c) haplotypes of SNPs in the “blocks”.

The effects of MARCO haplotypes on disease were estimated using four gamete rule as default set and accelerated expectation maximization (EM) algorithm as implemented in the program HaploView v3.2. A total of 4 blocks (figure 1) and 14 Haplotypes with frequencies greater than 1% were identified (Table 4). Of 4 constructed blocks, neither of the haplotypes involved in block 1 (rs6752783 T/G and rs7559955 C/T) had significant association with susceptibility to pTB (table 2). However, both GC haplotype involved in block 2 (rs17009726A/G, rs2278588C/T) and TGCC haplotype in block 3 (rs17795618T/A, rs1371562G/T, rs6761637T/C, rs2011839C/T) were associated with an increased pTB risk after 100000 permutations analysis using haploview (*p*_corrected_ = 0.0001 and 0.0297, respectively; table 2). No other significant differences were observed in haplotypes frequencies involved in block4 between two groups.

**Table 4:** MARCO haplotypes analysis in pulmonary tuberculosis cases and controls.

Haplotype	Case	Control	*p*-value	*p*_corrected_-value*
**Block 1**				
**TC**	0.771(1423.6)	0.772(1594.5)	0.964	1.000
**GT**	0.188(347.6)	0.205(424.5)	0.178	0.870
**TT**	0.026(47.4)	0.018(37.5)	0.106	0.728
**Block 2**				
**AC**	0.617(1139.3)	0.654(1350.3)	0.018	0.199
**AT**	0.244(449.7)	0.254(525.7)	0.435	0.998
**GC**	0.136(250.7)	0.089(183.7)	**3.13E-6**	**0.0001**
**Block 3**				
**TGTC**	0.527(973.6)	0.534(1103.5)	0.674	1.000
**AGTC**	0.189(348.2)	0.205(424.1)	0.192	0.904
**TGCC**	0.148(272.7)	0.114(235.3)	**0.0017**	**0.0297**
**TTTC**	0.084(155.5)	0.090(186.5)	0.506	0.999
**TTTT**	0.020(36.6)	0.029(59.9)	0.065	0.536
**Block 4**				
**GCT**	0.569(1048.5)	0.563(1162.5)	0.711	1.000
**GTT**	0.242(446.5)	0.259(534.5)	0.234	0.941
**GCC**	0.144(265.6)	0.136(281.4)	0.479	0.999
**TCT**	0.040(73.9)	0.035(73.1)	0.443	0.998

Data are presented as frequency of haplotype (n), unless otherwise stated.

*p value obtained from haplotype analysis after the 100,000 permutations testing.

Considering the association with a genetic variant and the pulmonary disease is too uncertain, we further explored the difference between patients with and without G allele of rs17009726 including age, sex, smoking, BCG status and TST, no significant difference was observed (table S1).

## Discussion

We have identified genetic variants in the MARCO gene which might be associated with increased susceptibility to pTB. Significant association results were observed for rs17009726 by single point analysis and, 2 SNPs GC haplotype (based on rs17009726 and rs2278588) and 4 SNPs TGCC haplotype (based on rs17795618, rs1371562, rs6761637 and rs2011839). On the other hand, No significant associations were observed for other SNPs.

When the previous studies have indicated that substantial genetics components have contributed to the susceptibility of TB [2], [4], a serial studies including population based and family based case control studies as well as linkage studies were performed across the different ethnic population, but the progress in determination of contributing genetic variants of pTB has been slow. The recently genome-wide association study (GWAS) performed in African population identified a pTB related single locus (rs4331426) on chromosome 18q11.2 with a modest effect size (OR = 1.2) [16]. However, unlike the GWAS for other infectious disease [17], [18], [19], [20], [21], the rs4331426 is within a gene-desert and its biologic mechanism is unknown. Therefore, identifying the new candidate genes involving *M.tb* infection may be helpful for understanding the host susceptibility to pTB.

MARCO gene in human is located on chromosome 2q12-q13. MARCO, as one of class A SRs, has been demonstrated as involving host defense against bacterial pathogens and requiring for TLR-signaling in macrophage response to *M.tb*. Given the crucial role of MARCO in host immunity against *M.tb* infection, we firstly investigated its SNPs and identified that the SNPs in MARCO were associated with increased risk to pTB in Chinese Han population.

Our data indicated that the G allele of rs17009726 alone or the haplotype GC may confer increased susceptibility to pTB insofar as both allele and haplotype frequencies are higher in pTB case subjects than in control subjects. As for rs6761637, the C allele or CC genotype was not conferred susceptibility to pTB in single-point analysis after multiple testing. When haplotypes were considered, haplotype TGCC containing C allele of rs6761637 were nominal associated with increased susceptibility to pTB and the haplotype frequencies are slightly higher in pTB case subjects than in control subjects. According to our study, the reasonable explanation for the genetic variants of MARCO associated with increased the susceptibility to pTB would be that the genetic variants could affect the expression of MARCO by influencing the mRNA expression or stability, which may alter the function of MARCO -expressing macrophages by decreasing bacterial binding and phagocytic capacity, as well as affecting the cooperation between MARCO and TLR2/CD14 and, less pro-inflammatory cytokines (TNF-a, IL-6, and IL-1β) production in response to *M.tb* infection.

One important consideration in addressing the significant genetic association is population stratification inherent to case control study, which however, could be minimized in the current research, as little subpopulation structure was observed for genotype distribution within northern Chinese populations [22]. Despite of this main advantage, the current research still lacked the independent replication in other ethnic populations. In addition, SNPs in the study were selected by searching the International HapMap project database, instead of by gene-wide resequencing of MARCO in Chinese Han population. This SNP tagging strategy might miss functional genetic variants, thus only common genetic variants could be implicated in understanding their roles in host susceptibility to or in progression of tuberculosis disease. The SNP identified to be associated with the pTB risk in the current study was located in the intron of the MARCO gene. According to the previous studies, intronic SNP located in the consensus 5′ splice site adjacent to an exon could cause the absence of exon or mutation due to the intron without splicing, and then affect the disease susceptibility. The bioinformatics analysis predicted no obvious function of this SNP in regulating transcription or translation of MARCO gene (data not shown). Therefore, functional assay of this SNP was not performed in the current stage, although this knowledge might help to get comprehensive understanding of the gene's role in affecting disease susceptibility.

Another important limit in our study is absence of knowledge about real latent TB infection, or contact with *M.tuberculosis* of the NHS who is presented as resistant to the disease. Although Chinese adults have been highly exposed to *M. tuberculosis*, we cannot verify the recent exposure to the bacteria of the NHS individually. This limit might lead to the misclassification of the group, which bias should be addressed. In addition, due to the lack of detailed information of the patients (the disease severity, anti-bacterial effect of the treatment, the TST results, et al), we failed to evaluate the association between the genetic variants and other characteristics of the patients, which makes this association less certain in the disease development.

Overall, we demonstrated for the first time that MARCO variants were strongly associated with increased risk to pTB in Chinese Han population. Further replication studies in other ethnic cohorts will be necessary to get comprehensive understanding of the functional effects of the identified genetic variants on pTB susceptibility.

## Materials and Methods

### Ethics Statement

The study was approved by ethics committee of the Hebei Chest Hospital, Shijiazhuang Fifth Hospital and Beijing Electronic Hospital. Written informed consent was obtained from all the participants before inclusion in the study.

### Study subjects

A total of 923 patients with pTB who were Chinese Han origin were recruited from the Hebei Chest Hospital and Shijiazhuang Fifth Hospital (Hebei, China) during March 2007 to August 2009. pTB patients were recruited by review of medical records and laboratory reports. Patients were diagnosed according to the diagnostic criteria for pulmonary tuberculosis of Ministry of Health, the People's Republic of China(http://www.moh.gov.cn/publicfiles///business/cmsresources/zwgkzt/cmsrsdocument/doc3242.pdf). The patients were defined as presence of at least one of the followings: (1) smear/culture positive for *M.tb* (2) culture positive for *M.tb* and pathological change of tuberculosis in lung according to chest X-ray (3) pathological change of tuberculosis in the lung according to of chest X-ray, typical clinical syndrome (4) pathological change of tuberculosis in the lung, culture positivity of bronchial lavage and/or pleural fluid for *M.tb*; (5) pathological change in the lung and pathological evidence of TB disease in lung biopsy materials (Lung tissue or tumor location). 1033 normal healthy subjects (NHS) who underwent the physical examination in the Beijing Electronic Hospital (Beijing, China) were recruited during the study period and were matched for age, sex, ethnic and geographical origins?to patients. The inclusion criteria for NHS were, absence of clinical signs, symptoms and pulmonary lesions on chest radiographic examination suggesting active tuberculosis. No medical history of TB or other infectious diseases, autoimmune disease, cancer or other disease that affect host immunity.

### SNPs selection and Genotyping

The SNPs tested were haplotype-tagging SNPs (htSNPs) in the MARCO gene based on the HapMap reference data (www.hapmap.org, the International HapMap Consortium 2003, release 22, on NCBI B36 assembly, dbSNP b126) for Chinese Han populations. SNPs were selected to serve as multimarker tagging algorithm with criteria of *r* ^2^>0.8 and for all SNPs with minor allele frequency (MAF) ≥5% using HaploView v3.2 program [23]. The entire MARCO gene was covered. Finally, 17 SNPs, including 14 SNPs in intron region (rs2077344 C/T, rs6752783 T/G, rs4491733 A/G, rs7559955 C/T, rs17009726 A/G, rs2278588 C/T, rs17795618 T/A, rs1371562 G/T, rs6751745 C/T, rs2011839 C/T, rs3731611 G/T, rs12987402 T/C, rs4848530 C/T, rs11685286 T/C), 1 SNP in exon (rs6761637 T/C), as well as 2 SNPs in promoter region (rs17009242 G/A, rs1318645 C/G) were selected (table 2).

Genomic DNA was isolated by Gene Relax DNA extraction kit (TianGen, Beijing, China) according to the manufacturer's instructions. Genotyping of SNPs was performed using the Sequenom system (Sequenom, San Diego, CA 92121-1331, USA) which uses mass spectrometry (MALDI-TOF) to discriminate products by their absolute masses. Primer extension was carried out utilizing a DNA primer adjacent to the SNP, and a specific reaction mix of polymerase, dNTPs and one ddNTP. Samples were analyzed by MALDI-TOF mass spectrometry and the alleles were called by weight (in daltons) of the extension products. Samples from positive and negative control subjects were included on each genotyping plate and checked for consistency. For confirmation the genotyping of variants, re-genotyping was verified by 10% replication of samples and by direct sequencing.

### Statistical analysis

The homogeneity of baseline characteristics between two groups was tested by Fisher's exact test or χ^2^ test for categorical variables and by Student's t-test or Wilcoxon test for continuous variables. Power calculation was performed with the CATS software (available at http://www.sph.umich.edu/csg/abecasis/CaTS/). The tests for deviations from Hardy–Weinberg equilibrium (HWE) were performed for each SNP by HaploView v3.2 program [23]. We used binary logistic regression to estimate the genotype-specific odds ratio (OR) and 95% confidence interval (CI) for pTB risk, after adjusting for sex, age, smoking and BCG vaccination status. An allelic *p* value of less than 0.05 was considered nominally significant and Bonferroni correction for multiple testing of 17 SNPs was applied to the single-point results of the genetic model analysis of genotype. Haplotype blocks were constructed using four gamete rule as default set and haplotype frequencies were inferred from the genotype data in the combined population (cases and controls) using an accelerated expectation maximization (EM) algorithm as implemented in the program HaploView v3.2. HaploView program was also used to evaluate the statistical significance of haplotype frequency differences between cases and controls with 100,000 permutations for multiple testing. Data were analyzed using Statistical Package for Social Sciences 17.0 (SPSS Inc, Chicago, IL, USA) and HaploView v3.2. In all analysis, statistical tests were based on two-tailed probability.

## Supporting Information

Table S1 **Characteristic comparison of patients with and without G allele of rs17009726.**
(DOC)
