# Supplementary material for: Genetic Variants in MARCO Are Associated with the Susceptibility to Pulmonary Tuberculosis in Chinese Han Population
Source: PLoS One. 2011 Aug 23;6(8):e24069. doi: 10.1371/journal.pone.0024069 (PMC3160327; doi:10.1371/journal.pone.0024069)
Supplement: Table S1 — Characteristic comparison of patients with and without G allele of rs17009726. (DOC) [file pone.0024069.s001.doc]

**Table S1 characteristic comparison of patients with and without G allele of rs17009726**

| Characteristics | non G allele | G allele | *p*-value |
| --- | --- | --- | --- |
| **Sex** |  |  | 0.17 |
| Female | 242(35.3) | 72(30.4) |  |
| Male | 444(64.7) | 165(69.6) |  |
| **Age yrs** | 36.9(16.7) | 34.9(16.4) | 0.11 |
| **Smoking** |  |  | 0.78 |
| Smoking | 153(22.0) | 47 (20.6) |  |
| Nonsmoking | 512(73.7) | 169(74.1) |  |
| Ever smoking | 30(4.3) | 12(5.3) |  |
| **BCG vaccination** |  |  | 0.82 |
| Yes | 140(20.9) | 45(19.4) |  |
| No | 496(73.9) | 173(74.6) |  |
| Uncertain | 35(5.2) | 14(6.0) |  |
| **Tuberculin skin test** |  |  | 0.66 |
| Positive | 373 (54.7) | 123(51.5) |  |
| Strong positive | 238(34.9) | 94(39.3) |  |
| Negative | 34(5.0) | 10(4.2) |  |
| ND | 37(5.4) | 12(5.0) |  |

Data are presented as n (%) or Mean (SD), unless otherwise stated.
